# Quantification of the variation in percentage identity for protein sequence alignments

**DOI:** 10.1186/1471-2105-7-415

**Published:** 2006-09-19

**Authors:** GPS Raghava, Geoffrey J Barton

**Affiliations:** 1 School of Life Sciences Research, University of Dundee, Dow Street, Dundee, DD1 5EH, Scotland, UK; 2 Bioinformatics Centre, Institute of Microbial Technology, Sector 39A, Chandigarh, India; 3 This work was intitated when both authors were at the University of Oxford, Laboratory of Molecular Biophysics, Rex Richards Building, Oxford, OX1 3QU, UK

## Abstract

**Background:**

Percentage Identity (PID) is frequently quoted in discussion of sequence alignments since it appears simple and easy to understand. However, although there are several different ways to calculate percentage identity and each may yield a different result for the same alignment, the method of calculation is rarely reported. Accordingly, quantification of the variation in PID caused by the different calculations would help in interpreting PID values in the literature. In this study, the variation in PID was quantified systematically on a reference set of 1028 alignments generated by comparison of the protein three-dimensional structures. Since the alignment algorithm may also affect the range of PID, this study also considered the effect of algorithm, and the combination of algorithm and PID method.

**Results:**

The maximum variation in PID due to the calculation method was 11.5% while the effect of alignment algorithm on PID was up to 14.6% across three popular alignment methods. The combined effect of alignment algorithm and PID calculation gave a variation of up to 22% on the test data, with an average of 5.3% ± 2.8% for sequence pairs with < 30% identity. In order to see which PID method was most highly correlated with structural similarity, four different PID calculations were compared to similarity scores (Sc) from the comparison of the corresponding protein three-dimensional structures. The highest correlation coefficient for a PID calculation was 0.80. In contrast, the more sophisticated Z-score calculated by reference to randomized sequences gave a correlation coefficient of 0.84.

**Conclusion:**

Although it is well known amongst expert sequence analysts that PID is a poor score for discriminating between protein sequences, the apparent simplicity of the percentage identity score encourages its widespread use in establishing cutoffs for structural similarity. This paper illustrates that not only is PID a poor measure of sequence similarity when compared to the Z-score, but that there is also a large uncertainty in reported PID values. Since better alternatives to PID exist to quantify sequence similarity, these should be quoted where possible in preference to PID. The findings presented here should prove helpful to those new to sequence analysis, and in warning those who seek to interpret the value of a PID reported in the literature.

## Background

Statistical measures of sequence similarity are routinely applied to quantify the results of sequence database searches [1,2]. However, having identified an interesing similarity, it is common practice to quote a value of percentage sequence identity (PID) for the alignment. PIDs may also be used in estimating phylogenetic trees from multiple sequence alignments, but this application is not addressed in this paper. PIDs are also frequently used as a cutoff when removing redundancy from large sets of sequences. At first sight, PID is a simple to understand measure, but in fact there is no standard method to calculate the value. PID calculations reported in the literature include those derived by dividing the number of identical positions in the alignment by the number of aligned positions [3,4], dividing by the shortest sequence [5] or dividing by the length of the aligned region (aligned positions excluding overhangs) [6]. When a PID value is quoted, it is rare for the method of calculation to be reported as well, but for the same alignment, each PID method may produce different values. Since PID is really a group of different similarity measures, it is important when interpreting a reported PID to know what variation in PID may be expected due to the differences in commonly used calculations. The value of PID is further complicated by the observation that different alignment techniques when applied to the same pair of sequences, or the same technique used with different parameters, may give alignments that show different values of PID. Together, these factors make it difficult to judge the significance of a PID value quoted for two protein sequences. This is a particular problem when comparing a PID value to general cutoffs for homology (e.g. [7].)

Recently, May[8] compared four different methods of calculating PID on a set of 9535 alignments derived from 3D-structure comparison to determine which were most similar. He concluded that dividing the number of identities by the mean sequence length gave PIDs that were most like PIDs calculated by other methods. However, May[8] did not explicitly report the range of PID observed for the same alignment, nor the effect of different alignment methods on the reported PID. Accordingly, in this paper we have examined the range of PID expected due to differences in the PID calculation method as well as the effect of different alignment techniques. Our analysis suggests which PID calculation is most robust, and as expected, indicates that PID by any method is a poor substitute for sequence comparison measures based on randomisation.

## Results

### Range of percentage identity seen for different PID calculations

Out of 1028 aligned pairs there were only 20 pairs where all four percentage identity measures had the same value. 711 pairs had differences in PID between 2% and 5%. There were 87 pairs for which the difference was greater than 5%. The greatest difference seen was 11.5%.

The difference between the maximum and minimum PID decreases slightly with increasing minimum PID. Thus, the average difference in PID for alignments with a minimum PID ≤ 30% was 3.3 ± 1.5 %, while the average difference for alignments with PID > 30% was 2.2 ± 1.5%.

PID2 was always largest since it considers only the aligned positions. PID4 was ≤ PID1 on most of the pairs. Differences between PID4 and PID1 were observed in pairs where one sequence overhangs at the N-terminal and other at the C-terminal. For most of the alignments, PID3 was higher than PID1 or PID4. PID4 gave slightly more consistent values of PID that were less prone to artefactually high or low values as a result of overhangs. PID4 also gave a slightly better correlation with structural similarity as shown in Table 1 and discussed below.

**Table 1:** Correlation between PIDs and structural similarity score. Z: Z-Score, (Also known as SD – Score) from randomisation.

Alignment	Weight Matrix	gap pen	PID1	PID2	PID3	PID4	Z	NAS
STAMP			0.85	0.82	0.84	0.86		
AMPS	BLOSUM62	10	0.79	0.76	0.77	0.80	0.84	0.82

NAS: Normalised Alignment Score, (see text for details).

### Range of percentage identity seen for different alignment methods

Ideally, one would calculate the PID between two sequences from the comparison of the protein three – dimensional structures. In the absence of structures for both proteins, sequence alignment techniques must be applied. Since alignment of sequences is an optimisation based on the parameters and algorithm, the resulting alignment depends on these factors. Accordingly, the range of PID4 was examined for the reference structural alignment and for sequence alignments obtained by the AMPS[9,10] alignment package with default parameters. For most pairs of sequences, the sequence alignment gave a higher PID4 than the reference alignment. This is to be expected, since the sequence alignment algorithm aims to produce an alignment that optimises sequence similarity, while the reference structural alignment is the result of an optimisation of structure comparison.

In order to understand the effect of the alignment algorithm on the PID, the same sequence pairs were aligned by AMPS [10], CLUSTALW [11] and GAP (GCG Version 9.1; which implements the Needleman & Wunsch, 1970 algorithm [12]) with default parameters. The difference in PID4 was between 0% and 14.6%. Most of the pairs had differences between 0% and 5%. A similar trend was observed for PID1, PID2 and PID3 (data not shown). One extreme example was the pair of domains linel-2 and 2hft-2 for which PID4 was 3.9% for the GAP alignment, 11.7 % for the CLUSTALW alignment and 18.5% for the AMPS alignment. However, none of these alignments agreed with the reference structural alignment. Overall, the difference in PID4 decreases with the increase in minimum PID4. Thus, the average difference for PID4 ≤ 30% was 2.5 ± 2.1% and > 30% was 0.73 ± 0.9%. This simply reflects the smaller dependency on parameters for alignments between sequences of higher similarity.

In the real-world situation where one is comparing PID values calculated in different ways by different algorithms, the results presented here suggest the range in PID difference will be between 0 and 21.8 %. The average difference for PID ≤ 30% was 5.3 ± 2.8% and > 30% was 2.7 ± 1.9%.

## Discussion

In this article it has been shown that the PID value was affected both by the way in which it was calculated, and by how the alignment was generated. While neither of these facts is particularly surprising, to our knowledge, this is the first time the range of PID has been reported for these effects. The combined effect of algorithm and calculation gave rise to differences in PID of up to 22%. Given these limitations, which PID calculation gave the most reliable estimate of similarity?

The STAMP structural comparison algorithm that was used to generate the reference alignments in this study provides a measure of structural similarity (Sc) which takes account of distance and conformational similarity, for each pair of proteins [13]. The correlation between Sc and PID1 to PID4 when calculated for the reference alignment is shown in Table 1. PID2 was the least correlated (*r *= 0.82), while PID4 was best correlated (*r *= 0.86), with PID1 marginally worse (*r *= 0.85). This suggests the order of reliability to be PID4 ≥ PID1 > PID3 > PID2. Table 1 also illustrates correlation values for PID1-4 for sequence alignments generated by AMPS [10] for the same pairs of proteins. Although the correlations are weaker, the trend is the same. Two further measures of sequence similarity were calculated for each pair of sequences. The Normalised Alignment Score (NAS) [9] was calculated by applying the BLOSUM62 matrix to the alignment, subtracting penalties for internal gaps, then dividing by the number of positions not aligned with gap. The Z-score was calculated by shuffling each sequence 100 times and comparing the shuffled sequences by the dynamic programming alignment algorithm [9]. The Z-score is given by:

Z-score=(V−x¯)σ     (1)
 MathType@MTEF@5@5@+=feaafiart1ev1aaatCvAUfKttLearuWrP9MDH5MBPbIqV92AaeXatLxBI9gBaebbnrfifHhDYfgasaacH8akY=wiFfYdH8Gipec8Eeeu0xXdbba9frFj0=OqFfea0dXdd9vqai=hGuQ8kuc9pgc9s8qqaq=dirpe0xb9q8qiLsFr0=vr0=vr0dc8meaabaqaciaacaGaaeqabaqabeGadaaakeaacqqGAbGwcqqGTaqlcqqGZbWCcqqGJbWycqqGVbWBcqqGYbGCcqqGLbqzcqGH9aqpdaWcaaqaaiabcIcaOiabdAfawjabgkHiTiqbdIha4zaaraGaeiykaKcabaacciGae83WdmhaaiaaxMaacaWLjaWaaeWaceaacqaIXaqmaiaawIcacaGLPaaaaaa@41A7@

where, V is the alignment score for two sequences, σ and x¯
 MathType@MTEF@5@5@+=feaafiart1ev1aaatCvAUfKttLearuWrP9MDH5MBPbIqV92AaeXatLxBI9gBaebbnrfifHhDYfgasaacH8akY=wiFfYdH8Gipec8Eeeu0xXdbba9frFj0=OqFfea0dXdd9vqai=hGuQ8kuc9pgc9s8qqaq=dirpe0xb9q8qiLsFr0=vr0=vr0dc8meaabaqaciaacaGaaeqabaqabeGadaaakeaacuWG4baEgaqeaaaa@2E3D@ are the mean and standard deviation of distribution of scores for shuffled sequences. The Z-score has the advantage over PID scores in that it corrects for the effect of alignment length and sequence composition. Z-scores for pair-wise sequence alignments may be converted to probabilities by applying the methods described by Webber & Barton[14]. The correlation value of 0.84 suggests the Z-score to be the best measure of similarity in the absence of a structural alignment, with NAS a close second.

## Conclusion

In this paper we have quantified the variation in reported percentage identity seen in 1028 structural alignments, due to different denominators in the PID calculation and due to alignment method. The overall conclusions are:

1. The four different PID denominators considered, gave up to 11.5% difference in PID on a single alignment in the test set.

2. Sequence alignments by three different methods resulted in variation of up to 14.6% PID on a single alginment in the test set.

3. Combination of PID calculation and alignment method led to variation of up to 22% PID on a single alignment in the test set.

4. PID calculations that take account of gaps (PID1 and PID4) were more highly correlated with the STAMP Sc score for structural similarity between the proteins, than those that do not consider gaps (PID2 and PID3).

5. All PID calculations were less well correlated with the STAMP Sc score than the Z-score obtained by comparison to shuffled sequence scores.

These overall conclusions are not surprising to those expert in sequence analysis, but to our knowledge this is the first time that the variation in PID has been quantified explicitly. Quantification of the variation in PID is valuable, since although PID is a poor substitute for more sophisticated scoring methods that take account of the physico-chemical properties of the amino acids and correct for sequence length, PID remains widely quoted. The findings presented here should prove helpful to those new to sequence analysis, and as a guide to those who seek to interpret the value of a PID reported in the literature.

## Methods

### Test data set

Protein domain families were taken from the OxBench database of reference alignments [15]. OxBench contains pair-wise and multiple sequence alignments for families of proteins of known three-dimensional structure. The alignment families in OxBench were selected by a process of automatic structural alignment followed by manual inspection and pruning. In this way, the structural alignments chosen for this study are likely to have higher confidence than alignments derived by a purely automatic procedure. In addition, highly similar sequences (PID3 > 70) and short alignments (shortest sequence < 100) were removed from the families. This left 1028 pairs of protein three-dimensional structures which were aligned by the STAMP structure comparison algorithm [13]. In order to remove any dependency on pre-existing sequence alignments, STAMP was run in scan mode to find the optimal starting transformation for each alignment.

### Calculation of percentage identity

For each reference structural alignment, the percentage identity was calculated in four different ways.

PID1 was calculated as described by Doolittle, (1981):

PID1=100(Identical PositionsAligned Positions+Internal Gap Positions)     (2)
 MathType@MTEF@5@5@+=feaafiart1ev1aaatCvAUfKttLearuWrP9MDH5MBPbIqV92AaeXatLxBI9gBaebbnrfifHhDYfgasaacH8akY=wiFfYdH8Gipec8Eeeu0xXdbba9frFj0=OqFfea0dXdd9vqai=hGuQ8kuc9pgc9s8qqaq=dirpe0xb9q8qiLsFr0=vr0=vr0dc8meaabaqaciaacaGaaeqabaqabeGadaaakeaacqqGqbaucqqGjbqscqqGebarcqaIXaqmcqGH9aqpcqaIXaqmcqaIWaamcqaIWaamdaqadiqaamaalaaabaGaemysaKKaemizaqMaemyzauMaemOBa4MaemiDaqNaemyAaKMaem4yamMaemyyaeMaemiBaWMaeeiiaaIaemiuaaLaem4Ba8Maem4CamNaemyAaKMaemiDaqNaemyAaKMaem4Ba8MaemOBa4Maem4CamhabaGaemyqaeKaemiBaWMaemyAaKMaem4zaCMaemOBa4MaemyzauMaemizaqMaeeiiaaIaemiuaaLaem4Ba8Maem4CamNaemyAaKMaemiDaqNaemyAaKMaem4Ba8MaemOBa4Maem4CamNaey4kaSIaemysaKKaemOBa4MaemiDaqNaemyzauMaemOCaiNaemOBa4MaemyyaeMaemiBaWMaeeiiaaIaem4raCKaemyyaeMaemiCaaNaeeiiaaIaemiuaaLaem4Ba8Maem4CamNaemyAaKMaemiDaqNaemyAaKMaem4Ba8MaemOBa4Maem4CamhaaaGaayjkaiaawMcaaiaaxMaacaWLjaWaaeWaceaacqaIYaGmaiaawIcacaGLPaaaaaa@86D9@

PID2 only considers matched residues [3,4]:

PID2=100(Identical PositionsAligned Positions)     (3)
 MathType@MTEF@5@5@+=feaafiart1ev1aaatCvAUfKttLearuWrP9MDH5MBPbIqV92AaeXatLxBI9gBaebbnrfifHhDYfgasaacH8akY=wiFfYdH8Gipec8Eeeu0xXdbba9frFj0=OqFfea0dXdd9vqai=hGuQ8kuc9pgc9s8qqaq=dirpe0xb9q8qiLsFr0=vr0=vr0dc8meaabaqaciaacaGaaeqabaqabeGadaaakeaacqqGqbaucqqGjbqscqqGebarcqaIYaGmcqGH9aqpcqaIXaqmcqaIWaamcqaIWaamdaqadiqaamaalaaabaGaemysaKKaemizaqMaemyzauMaemOBa4MaemiDaqNaemyAaKMaem4yamMaemyyaeMaemiBaWMaeeiiaaIaemiuaaLaem4Ba8Maem4CamNaemyAaKMaemiDaqNaemyAaKMaem4Ba8MaemOBa4Maem4CamhabaGaemyqaeKaemiBaWMaemyAaKMaem4zaCMaemOBa4MaemyzauMaemizaqMaeeiiaaIaemiuaaLaem4Ba8Maem4CamNaemyAaKMaemiDaqNaemyAaKMaem4Ba8MaemOBa4Maem4CamhaaaGaayjkaiaawMcaaiaaxMaacaWLjaWaaeWaceaacqaIZaWmaiaawIcacaGLPaaaaaa@697F@

PID3 only considers the shortest sequence [5] :

PID3=100(Identical PositionsShortest Sequence)     (4)
 MathType@MTEF@5@5@+=feaafiart1ev1aaatCvAUfKttLearuWrP9MDH5MBPbIqV92AaeXatLxBI9gBaebbnrfifHhDYfgasaacH8akY=wiFfYdH8Gipec8Eeeu0xXdbba9frFj0=OqFfea0dXdd9vqai=hGuQ8kuc9pgc9s8qqaq=dirpe0xb9q8qiLsFr0=vr0=vr0dc8meaabaqaciaacaGaaeqabaqabeGadaaakeaacqqGqbaucqqGjbqscqqGebarcqaIZaWmcqGH9aqpcqaIXaqmcqaIWaamcqaIWaamdaqadiqaamaalaaabaGaemysaKKaemizaqMaemyzauMaemOBa4MaemiDaqNaemyAaKMaem4yamMaemyyaeMaemiBaWMaeeiiaaIaemiuaaLaem4Ba8Maem4CamNaemyAaKMaemiDaqNaemyAaKMaem4Ba8MaemOBa4Maem4CamhabaGaem4uamLaemiAaGMaem4Ba8MaemOCaiNaemiDaqNaemyzauMaem4CamNaemiDaqNaeeiiaaIaem4uamLaemyzauMaemyCaeNaemyDauNaemyzauMaemOBa4Maem4yamMaemyzaugaaaGaayjkaiaawMcaaiaaxMaacaWLjaWaaeWaceaacqaI0aanaiaawIcacaGLPaaaaaa@69B5@

PID4 considers the shortest length (sequence plus gap positions).

PID4=(Identical Positionsmin⁡(TGA,TGB))     (5)
 MathType@MTEF@5@5@+=feaafiart1ev1aaatCvAUfKttLearuWrP9MDH5MBPbIqV92AaeXatLxBI9gBaebbnrfifHhDYfgasaacH8akY=wiFfYdH8Gipec8Eeeu0xXdbba9frFj0=OqFfea0dXdd9vqai=hGuQ8kuc9pgc9s8qqaq=dirpe0xb9q8qiLsFr0=vr0=vr0dc8meaabaqaciaacaGaaeqabaqabeGadaaakeaacqqGqbaucqqGjbqscqqGebarcqaI0aancqGH9aqpdaqadiqaamaalaaabaGaemysaKKaemizaqMaemyzauMaemOBa4MaemiDaqNaemyAaKMaem4yamMaemyyaeMaemiBaWMaeeiiaaIaemiuaaLaem4Ba8Maem4CamNaemyAaKMaemiDaqNaemyAaKMaem4Ba8MaemOBa4Maem4CamhabaGagiyBa0MaeiyAaKMaeiOBa4MaeiikaGIaemivaqLaem4raC0aaSbaaSqaaiabdgeabbqabaGccqGGSaalcqWGubavcqWGhbWrdaWgaaWcbaGaemOqaieabeaakiabcMcaPaaaaiaawIcacaGLPaaacaWLjaGaaCzcamaabmGabaGaeGynaudacaGLOaGaayzkaaaaaa@5E34@

Where *TG*_*A *_and *TG*_*B *_are the sum of the number of residues and internal gap positions in sequences *A *and *B *in the alignment.

In this study, all PID values were calculated over the complete alignment rather than the structurally conserved core. This reflects the situation when aligning two protein sequences where neither protein has a known three-dimensional structure and so the structurally conserved core is unknown.

## Authors' contributions

GPSR ran the programs, wrote analysis code and drafted the paper. GJB conceived and directed the project, and finalised the analysis and manuscript.
